# Prevalence of Hemoglobinopathies Among the Kurdish Population in Zakho City, Kurdistan Region, Iraq

**DOI:** 10.7759/cureus.78862

**Published:** 2025-02-11

**Authors:** Shakir Abdulrahman Zebari

**Affiliations:** 1 Hematopathology, College of Medicine, University of Zakho, Zakho, IRQ

**Keywords:** beta-thalassemia minor, hemoglobinopathy, iraq, kurdistan region, prevalence, sickle cell trait, zakho

## Abstract

Background: Hemoglobinopathies are the most common inherited disorders in the Mediterranean region, including Iraq, with different frequencies and molecular features. They represent an important health problem in Iraq. Several studies have addressed the prevalence of hemoglobinopathy in Iraq, but no specific research has addressed its prevalence in Zakho city, Kurdistan Region, Iraq. Thus, this study was conducted to address this issue.

Materials and methods: A prospective cross-sectional study was conducted in a premarital screening center at Zakho Emergency Teaching Hospital, involving 11,910 Kurdish persons attending premarital screening investigations. Blood samples were collected from all participants for complete blood counts and sickling tests. A cut-off value of hemoglobin (Hb) concentrations (<13.0 gm/dl in males, <12.0 gm/dl in females), mean cell volume (MCV) <80 fl and/or mean cell hemoglobin (MCH) <27 pg, and the results of the sickling test were used to categorize the participants into three groups: group I, normal (normal Hb, MCV, and MCH, and negative sickling test); group II, individuals with MCV < 80 fl and/or MCH < 27 pg, with or without anemia, and negative sickling test; group III, sickle positive (individuals with a positive sickling test, with or without anemia, regardless of MCV and/or MCH values).

Groups II and III underwent further evaluations by morphology, reticulocyte count, hemoglobin-H preparation, iron studies, and cation exchange high-performance liquid chromatography (HPLC). β-thalassemia trait was diagnosed by hypochromic microcytic red cell indices and increased hemoglobin A2 (HbA2) (>3.5%). The sickle cell trait was diagnosed by the presence of a band at the S region at HPLC with a positive sickling test. Iron deficiency anemia (IDA) was diagnosed by hypochromic microcytic anemia and reduced transferrin saturation (<15%) and/or ferritin (<15 mg/L). Descriptive statistical analyses were conducted to define significant differences between the study's findings.

Results: A total of 963 (8.09%) of the studied participants were in group II. Further investigations on these 963 cases confirmed a diagnosis of IDA in 610 (5.12%). β-thalassemia trait was detected in 308 cases, with an overall prevalence of 2.59%. δβ-thalassemia trait was detected in nine (0.076%) of the studied participants. A professional diagnosis of α-thalassemia was given to 36 (0.30%) of those with hypochromic microcytic red cell indices, normal iron studies, and HPLC results. A positive sickling test was seen in 48 participants and confirmed by HPLC as a sickle cell trait with a prevalence of 0.4%. The consanguinity rate among couples was 28.8%. Neither case of HbC or HbD trait, nor combined hemoglobinopathy, was detected in the current study. Furthermore, no differences were found between the prevalence of both β thalassemia and sickle cell trait with either sex and/or religion, with a p-value of 0.07, 0.07, 0.532, and 0.78, respectively.

Conclusion: The prevalence of hemoglobinopathy in Zakho city is lower than in other regions of Iraq but still constitutes a significant burden on health services and financial resources. Careful interpretations of red cell indices, together with intensified premarital screening and counseling programs, coupled with an urgent need to increase awareness about the effects of consanguinity marriages on the prevalence of autosomal recessive disorders, can further reduce the incidence of hemoglobinopathies.

## Introduction

Hemoglobinopathies are among the common autosomal recessive diseases affecting humans [1]. They are categorized into the globin chains' qualitative and/or quantitative defects [1]. Thalassemia syndromes are characterized by quantitative defects in the alpha and beta genes, leading to α- and β-thalassemia, respectively [2]. Qualitative or structural abnormalities include hemoglobin S (HbS), hemoglobin E (HbE), hemoglobin C (HbC), and hemoglobin D (HbD) [2].

The incidence of the β-thalassemia trait is notably high in the Mediterranean, Southeast Asia, South China, the Middle East, and the Indian regions [3]. Approximately 7% of the global population carries β-thalassemia gene mutations, making hemoglobinopathies a significant global health concern, especially in developing countries like Iraq [2]. It was found that hemoglobinopathies occur worldwide, with significantly varied prevalence from one country to others and even between different regions within a country. Additionally, with population migration, the prevalence of these disorders is gradually increasing across different areas [2].

In almost all Arab countries, including Iraq, β-thalassemia has polymorphic variation, with carrier rates detected in 1% to 11% [4]. β-thalassemia in its heterozygous state is mostly asymptomatic, and characterized by normal to mild degree anemia, hypochromic microcytic red cell indices, and increased hemoglobin A2 (HbA2) level to levels between 3.5% and 7.0%, with normal or mildly elevated hemoglobin F (HbF). In contrast, compound heterozygosity and homozygosity (β-thalassemia major) classically present with severe hypochromic microcytic anemia and are dependent on regular transfusion for survival, with consequences of iron overload unless bone marrow transplantation is successfully undertaken, and this constitutes a significant health issue in Arab countries, including Iraq [2,4]. β-thalassemia intermedia lies between these two extremities with varied clinical and laboratory presentations due to genetic modifiers affecting either homozygous or heterozygous β-thalassemia [5].

Similarly, α-thalassemia is widely prevalent in Arab countries with frequencies ranging from 1% to 58% [4]. α- thalassemia has varied clinical and laboratory features ranging from a silent carrier with a single alpha-globin gene deletion to severe hydrops fetalis with all four alpha-globin gene deletions [2,5]. Two alpha globin gene deletions and three alpha globin deletions were presented with α-thalassemia minor and hemoglobin-H disease, respectively, with varied presentation from an asymptomatic to mild or moderate anemia [5].

The HbS gene has historically reached high frequencies in malaria-endemic regions over many generations with a selective advantage against *Plasmodium falciparum* malaria. However, population migration over decades has contributed to the distribution of the HbS gene to non-endemic regions [2,4]. Sickle cell disorder is most common among people in Africa, India, the Caribbean, the Middle East, and the Mediterranean [2]. It is characterized by valine substitution by glutamic acid at position six of the β-globin gene and characterized by various phenotypes, from an asymptomatic carrier state (sickle cell trait) to severe, transfusion-dependent vaso-occlusive disorders [4,5].

Sickle cell disorders include sickle cell anemia (Hb SS genotype) and sickle cell syndrome, with various combinations of Hb S with other abnormal hemoglobins, including sickle beta-thalassemia syndromes (HbS-β0 thalassemia, HbS-β+thalassemia), hemoglobin SC (HbSC) disorder, hemoglobin SE (HbSE) syndrome, HbS/hereditary persistence of fetal hemoglobin (S/HPFH), and many other rare combinations [6].

In Iraq, β-thalassemia syndromes are uniformly distributed throughout the country, with an overall carrier rate of around 4% with over 15,000 registered thalassemia major/intermedia patients. Meanwhile, sickle cell disorders show variable carrier rates ranging from 0% to 16.0% and are mainly clustered in the north and south of Iraq [4]. The relatively high carrier rates of hemoglobinopathy, coupled with relatively very high rates of consanguineous marriages around 25-30% among couples of Iraqi populations [7,8], and the already limited resources to the health care sector, dictate the necessity of prevention and counseling programs.

Screening requires an accurate and precise method and an understanding of the prevalence of various hemoglobinopathy disorders across the country. Screening is usually carried out to detect the asymptomatic carriers among couples and typically involves a comprehensive evaluation of the clinical and family history [9,10]. The laboratory methods for carrier identification include red blood cell (RBC) parameters, a sickling test, and a peripheral smear examination. Confirmatory tests through various methods like globin chain analysis, DNA analysis, and high-performance liquid chromatography (HPLC) are the simplest, widely available method in many areas and speed and relatively low cost for detecting most β-globin chain variants [9-11].

Hemoglobinopathy is an important health problem, and there are >100 registered major hemoglobinopathies from Zakho city registered in the Jin Pediatric Hematology-Oncology Center in the Duhok Governorate near Zakho city. Although different studies address the prevalence of hemoglobinopathies in different parts of Iraq, there are no available data in our locality. Therefore, the aim of this study is to determine the prevalence of hemoglobinopathies among the Kurdish population attending a premarital screening in Zakho city, Kurdistan Region, Iraq.

## Materials and methods

This prospective cross-sectional study was conducted at a premarital screening center at Zakho Emergency Teaching Hospital between January 2, 2021, and January 31, 2024. The study included the first five couples who attended mandatory premarital screening daily, with a total of 11,910 participants included in this study.

Non-Kurdish populations and non-residents of Zakho city were excluded from this study. All participants were informed about the benefits and importance of premarital screening, especially regarding hemoglobinopathies and sexually transmitted diseases, and verbal informed consent was obtained from all participants. Sociodemographic data were collected, including names, date of birth, residency, degree of relationship with each other, family history of hemoglobinopathy, history of blood transfusion, time from engagement to marriage date, phone numbers, and photos of couples. These records were stored and kept in confidential files and accessed only by authorized premarital staff.

Five milliliters (ml) of blood was collected from each participant by proper phlebotomy techniques. The blood was evenly distributed into a K3 ethylenediaminetetraacetic acid (EDTA) vacutainer tube, well mixed on a rotatory mixer, and a plain vacutainer tube, which is separated to obtain the serum and stored for later use of iron study when needed.

The EDTA-anticoagulated blood samples were analyzed for complete blood count (CBC) and sickling test. The CBC was performed using the Sysmex XN-550 fully automated hematology analyzer (Sysmex Corporation, Kobe, Japan) in reticulocyte (RET) mode. The hemoglobin (Hb) concentration in gram/deciliter (gm/dl), red blood cell (RBC) count (*1012/L), mean cell volume (MCV) in femtoliter (fl), mean cell hemoglobin (MCH) in picogram (pg), red cell distribution width (RDW) percentage (%), and reticulocyte percentage (%) were recorded for all participants.

The sickling test was performed for all participants, in which a drop of well-mixed EDTA blood was placed between a glass slide and coverslip, sealed with nail varnish, and incubated at 37°C for 24 hours. A microscopic examination was performed to detect the sickle cells. If HbS is present, the red cells lose their smooth, round shape and become sickled [11].

HPLC was performed using the Bio-Rad D-10^TM^ fully automated HPLC system (Bio-Rad Laboratories, Hercules, CA) for all cases with a positive sickling test as well as for individuals with an MCV < 80 fl and/or MCH < 27 pg, with or without anemia (Hb = 13.0 g/dl for males and Hb = 12.0 gm/dl for females used as a cut-off value for anemia). The proportions (%) of normal and/or abnormal Hb fractions were documented [2,11,12].

Iron studies, including serum iron, total iron-binding capacity, calculation of transferrin saturation (TS%), and ferritin levels, were performed for cases with MCV < 80 fl and/or MCH < 27 pg, or anemia (Hb < 12.0 gm/dl for females and Hb < 13.0 gm/dl for males). These tests were conducted using a chemiluminescence method on the Cobas c311 fully automated biochemistry analyzer (Roche Diagnostics, Basel, Switzerland) [11,13].

Participants were categorized into three groups based on the presence of anemia (Hb < 12.0 gm/dl for females, Hb < 13.0 gm/dl for males), MCV < 80 fl and/or MCH < 27 pg, and the sickling test results. Group I (normal) included individuals with normal Hb, MCV, and MCH, and a negative sickling test. Group II included individuals with MCV < 80 fl and/or MCH < 27 pg, with or without anemia, and a negative sickling test. This group underwent further evaluation by morphology (Giemsa stain), iron studies, hemoglobin-H (Hb-H) preparation, and HPLC analysis [11]. Group III (sickle positive) included individuals with a positive sickling test, with or without anemia, regardless of the MCV and/or MCH values. This group underwent further evaluation using morphology (Giemsa stain), iron studies, and confirmatory HPLC analysis [11].

Based on the results of complete blood count, iron studies, and HPLC analysis, individuals in group II were categorized into four major categories: iron deficiency anemia (IDA), β-thalassemia trait, δβ-thalassemia trait, and a provisional diagnosis of α-thalassemia. IDA is diagnosed in individuals with hypochromic microcytic anemia, with a cut-off value of 15 ng/ml for serum ferritin, and/or transferrin saturation <15% [11,13]. β-thalassemia trait is considered in individuals with hypochromic microcytic red cell indices, with an HbA2 level greater than 3.5% [2,11,12]. δβ-thalassemia trait is considered in individuals with hypochromic microcytic red cell indices, normal/low HbA2 level, with an increased HbF level (5-20%) [2,11,12]. A provisional diagnosis of α-thalassemia is given to individuals with hypochromic microcytic red cell indices, normal/low HbA2 and HbF levels, and normal iron studies. Genetic and family studies were recommended for confirmation [2,11,12].

Statistical method

Descriptive statistical methods were performed using IBM SPSS version 25 (IBM Corp., Armonk, NY). Categorical variables were expressed as frequencies and percentages, while continuous variables were expressed as mean ± standard deviation (SD). Fisher's exact test with a p-value of <0.05 was considered statistically significant.

Ethical approval

This study was approved by the Research Ethics Committee, College of Medicine, University of Zakho (Reference No.: DEC2020/UOZE34; issued date: 10/12/2020).

## Results

A total of 11,910 samples were analyzed during the study period, with 5,955 (50%) males and 5,955 (50%) females. The median age group for all participants was 25.0 years, with a mean age of 25.69 ± 6.04 years. Regarding religion, 11,786 participants (99%) were Muslims, 63 (0.5%) were Yezidis, and 59 (0.5%) were Christians.

The mean hemoglobin concentration for all participants was 13.72 ± 1.47. The mean RBC count was 4.88 ± 0.42, the mean MCV was 84.07 ± 8.04, the mean MCH was 28.19 ± 2.70, the mean RDW% was 12.80 ± 1.36, and the mean reticulocyte percentage was 1.1 ± 0.93.

The consanguinity rate was found to be 28.8% among couples (third and fourth-degree relatives). Regarding the time to marriage appointments, 3,965 couples (66.58%) had fewer than 14 days to wedding time, with the majority having less than seven days. Another 1,745 couples (29.30%) had between 14 and 30 days remaining, while 245 couples (4.12%) had more than 30 days to their marriage appointment.

Of all enrolled participants, 10,899 (91.51%) were found to have normal hemoglobin concentration, normal red cell indices, and negative sickling tests. These participants were categorized as group I (normal), and no further tests were performed. Meanwhile, 963 (8.09%) participants show low MCV and/or MCH with or without anemia and a negative sickling test. These participants were categorized as group II. While the remaining 48 (0.40%) of the analyzed samples showed a positive sickling test with or without anemia, regardless of MCV and/or MCH values. These participants were categorized as group III (sickle-positive).

**Figure 1:**
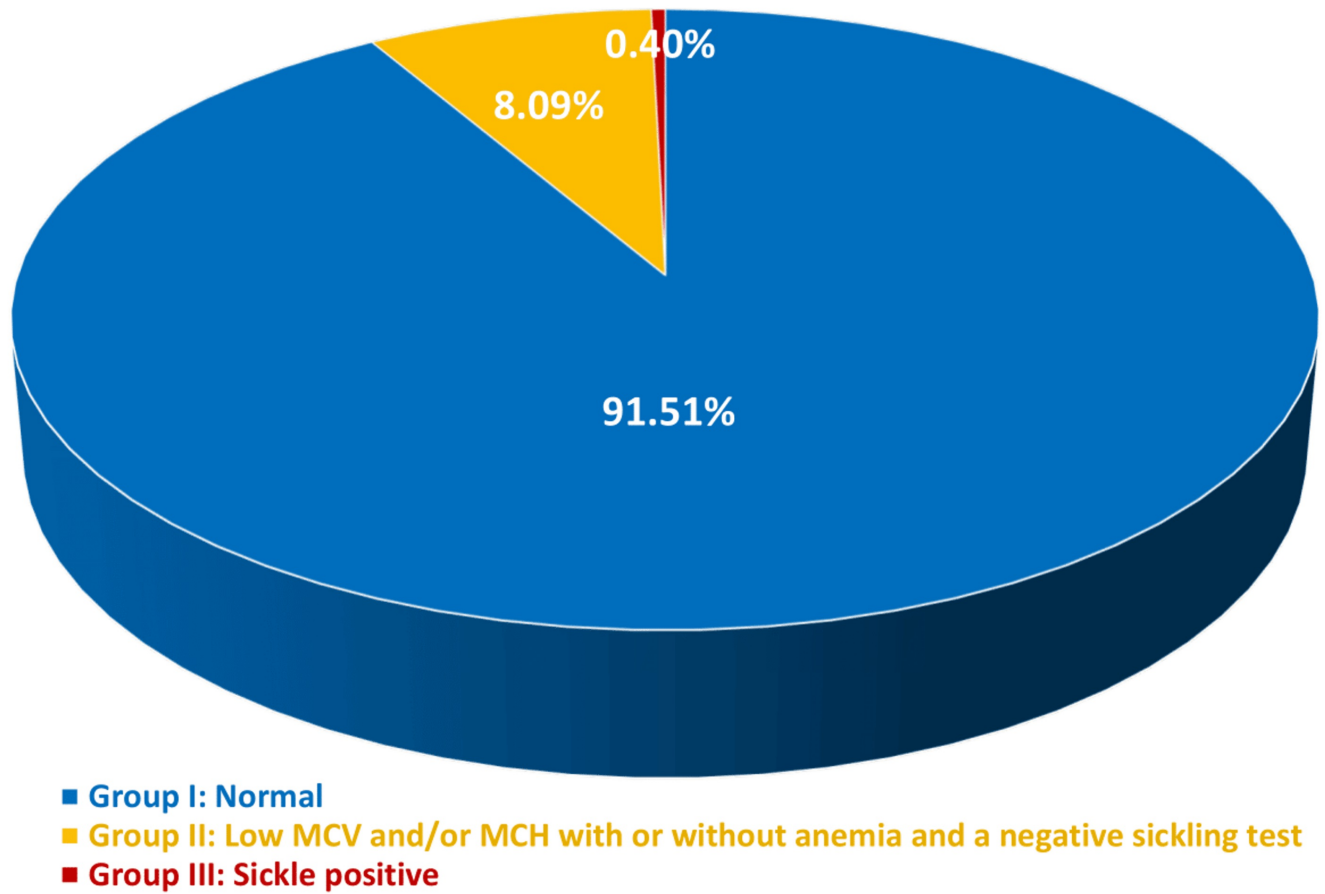
Distribution of participants (11,910 cases) into three groups according to hemoglobin concentration, red cell indices, and the results of the sickling test. Results are given as percentages of each group. MCV: mean cell volume; MCH: mean cell hemoglobin.

The percentages and the distribution of all participants into three groups are given in Figure 1.

A detailed description of group II and group III is explained below.

**Figure 2:**
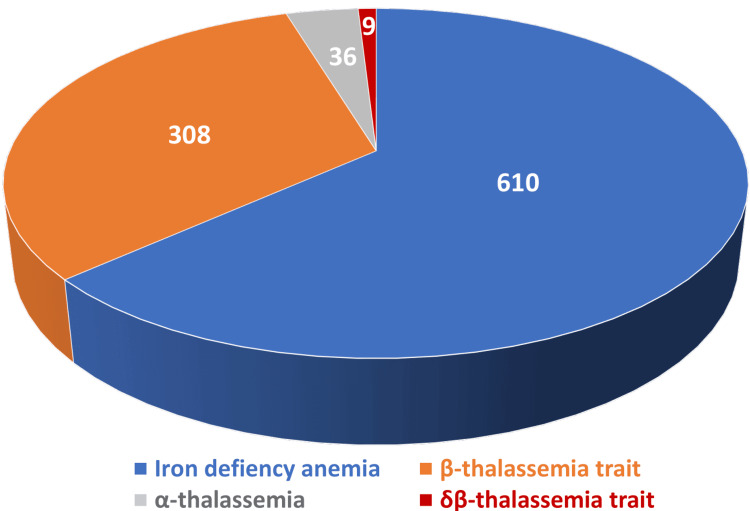
Distribution of group II into four categories based on the results of complete blood counts, iron studies, and HPLC analysis. The results are given as numbers for each category. HPLC: high-performance liquid chromatography.

Group II, i.e., low MCV and/or MCH, with or without anemia and a negative sickling test, was observed in 963 participants (8.09%). Participants in this group underwent further investigations, including morphological examination, Hb-H preparation, iron studies, and HPLC analysis. Based on these evaluations, participants were distributed into four categories: IDA, β-thalassemia trait, δβ-thalassemia trait, and a provisional diagnosis of α-thalassemia. The distribution of these categories is shown in Figure 2.

Iron deficiency anemia

IDA was identified in 610 cases, with an overall prevalence of 5.12%. The mean hemoglobin concentration was 9.40 ± 1.70, the mean RBC count was 3.9 ± 1.01, the mean MCV was 71.92 ± 2.80, the mean MCH was 22.80 ± 0.39, the mean RDW% was 16.20 ± 1.95, and the mean reticulocyte percentage was 0.4 ± 0.32. IDA was significantly detected in females than males (570 females versus 40 males), with a statistically significant p-value of 0.001. However, there were no statistically significant differences observed in the frequency of IDA with religion (p-value = 0.418) (Table 1).

**Table 1:** Demographic data, hemoglobin concentration, red cell indices, iron studies, and the result of HPLC analysis among group II. Results are given as mean ± SD and frequency/percentage as required. HPLC: high-performance liquid chromatography; Hb: hemoglobin; RBC: red blood cells; MCV: mean cell volume; MCH: mean cell hemoglobin; RDW: red cell distribution width; HbA2: hemoglobin A2; IDA: iron deficiency anemia.

Parameters	IDA	β-thalassemia trait without anemia	β-thalassemia trait with anemia	δβ-thalassemia trait	Provisional diagnosis of α-thalassemia
Hb (gm/dl)	9.40±1.70	13.1±0.6	12.1±0.2	10.40±0.40	10.70±0.90
RBC (*10^12^/L)	3.9±1.01	5.61±0.24	5.20±0.34	4.99±1.09	5.40±1.01
MCV (fl)	71.92±2.80	68.60±3.2	66.12±2.97	66.45±1.28	65.10±1.09
MCH (pg)	22.80±0.39	20.12±1.80	19.6±1.45	20.21±0.69	19.20±1.01
RDW (%)	16.20±1.95	13.20±0.60	13.40±1.10	15.12±1.29	16.10±2.40
Reticulocyte (%)	0.40±0.3	2.10±1.55	2.01±0.80	2.40±1.10	5.90±2.20
Ferritin (ng/dl)	7.01±2.19	64.23±3.83	59.12±4.22	62.12±1.96	49.09±1.30
Transferrin saturation (%)	8.11±1.97	37.12±1.20	30.40±2.35	48.12±2.90	37.97±3.29
HbA2 (%)	1.90±1.12	5.09±0.62	5.00±0.92	1.89±0.69	1.9±0.41
Hb F (%)	0.70±0.13	1.74±1.44	1.80±1.02	11.25±2.01	0.7±0.5
Hb S (%)	0	0	0	0	0
Males	40 (6.65%)	90 (29.23%)	40 (12.98%)	4(44.44%)	20 (55.55%)
Females	570 (93.44%)	18 (5.85%)	160 (51.94%)	5 (55.56%)	16 (44.45%)
Religion - Muslim	540 (88.52%)	165 (53.57%)	135 (43.84%)	9 (100%)	34 (94.44%)
Religion - Yezidi	40 (6.56%)	1 (0.32%)	1 (0.32%)	0	1 (2.78%)
Religion - Christian	30 (4.92%)	1 (0.32%)	5 (1.63%)	0	1 (2.78%)

β-thalassemia trait

The β-thalassemia trait was observed in 308 participants, with a prevalence of 2.59%. Among these, 108 cases (35.07%) had hypochromic microcytic red cell indices with normal hemoglobin concentrations for their age and sex, while 200 cases (64.93%) presented with hypochromic microcytic anemia. The condition was more commonly observed in females with anemia (160 females versus 40 males). Interestingly, no cases of combined β-thalassemia trait with sickle cell trait or IDA were observed. No statistically significant differences in the prevalence of β-thalassemia with sex or religion were observed, with p-values of 0.07 for both (Table 1).

δβ-thalassemia trait

The δβ-thalassemia trait was diagnosed in only nine participants, with an overall prevalence of 0.076%. All δβ-thalassemia traits diagnosed in this study presented with hypochromic microcytic anemia. Notably, combined δβ-thalassemia with sickle cell trait or IDA was not observed in any cases. The prevalence of δβ-thalassemia shows no statistically significant differences with either sex or religion, with p-values of 0.81 and 1.0, respectively (Table 1).

Provisional diagnosis of α-thalassemia

A provisional diagnosis of α-thalassemia was given to 36 participants (0.30%) based on findings of hypochromic microcytic red cell indices, a high RBC count, normal iron studies, and normal/low HbA2. Molecular and family studies were advised for final confirmation of the diagnosis. Notably, α-thalassemia was observed in males more than females (20 males versus 16 females), but the difference was statistically insignificant (p-value = 0.09). No statistically significant differences were observed in the prevalence of α-thalassemia with religion (p-value = 0.78) (Table 1).

Group III, sickle-positive

Positive sickling tests were detected in 48 participants (26 females versus 22 males), with an overall prevalence of 0.4%. This group underwent further evaluations, including morphological examination, iron studies, and HPLC analysis for confirmation. Of those with a positive sickling test, 24 participants (50%) were diagnosed with normochromic normocytic anemia, while the remaining 24 (50%) had normal blood count parameters (normal Hb, MCV, and MCH). The mean hemoglobin S (HbS%) for all positive cases was 35.1 ± 2.6, the mean hemoglobin F (HbF%) was 0.92 ± 0.5, and the mean hemoglobin (HbA2%) was 2.5 ± 0.4.

Despite the high prevalence of anemia in females with positive sickling test, no significant differences were observed in red cell indices, HbS%, HbF%, or HbA2% between those presented with and without anemia. Notably, no cases of combined sickle cell trait with β-thalassemia, α-thalassemia, or IDA were observed. Additionally, no cases of HbC or HbD were detected in this study. There were no statistically significant differences in the prevalence of sickle cell trait with either sex or religion, with p-values of 0.532 and 0.78, respectively (Table 2).

**Table 2:** Demographic data, hemoglobin concentration, red cell indices, iron studies, and the results of HPLC analysis among group III. Results are given as mean ± SD and frequency/percentage as required. HPLC: high-performance liquid chromatography; Hb: hemoglobin; RBC: red blood cells; MCV: mean cell volume; MCH: mean cell hemoglobin; RDW: red cell distribution width.

Parameters	Sickle cell trait with anemia	Sickle cell trait without anemia
Hb (gm/dl)	11.1±1.91	14.0±0.4
RBC (*10^12^/L)	4.6±1.01	4.85±0.42
MCV (fl)	84.1±3.4	85.60±4.21
MCH (pg)	28±1.2	29.9±2.2
RDW (%)	13.9±1.02	13.0±1.0
Reticulocyte (%)	1.9±0.12	2.0±0.29
Ferritin (ng/dl)	89±23	111±5.9
Transferrin saturation (%)	19.7±2.9	29±3.8
Males	4 (8.33%)	18 (37.5%)
Females	20 (41.66%)	6 (12.5%)
Religion - Muslim	6 (12.5%)	41 (85.42%)
Religion - Yezidi	0	0
Religion - Christian	1 (2.08%)	0

## Discussion

Premarital screening programs for hemoglobinopathies aim to identify asymptomatic carriers of these relatively common autosomal recessive disorders. These programs help couples understand the reproductive risks of having offspring of these preventable major hemoglobinopathies and explore their available options [14]. These screening programs form the basic preventive strategy for hemoglobinopathies, an approach adopted by the WHO program. Major hemoglobinopathies impose a significant burden on health resources and families and can only be cured by successful bone marrow transplantation [15].

Since the 1970s, premarital screening programs for the identification of couples at risk of hemoglobinopathies have been adopted in several countries where consanguinity and/or hemoglobinopathies are prevalent, such as Cyprus, Greece, the UK, and Italy, with evidence of success in reducing the incidence of affected birth rates [16]. The WHO recommends that such screening tests should be voluntary [17]. However, the success of these programs has led many countries in the Middle East, where hemoglobinopathies are a major health problem and highly prevalent, to adopt similar programs [16]. The Kurdistan region of Iraq adopted the mandatory premarital screening for marriage by law regulations. In addition to hemoglobinopathy screening, premarital screening also includes screening for ABO and Rh(D) blood groups, sexually transmitted diseases such as hepatitis B surface antigen, hepatitis C antibody, human immunodeficiency virus antibody, and Trypanosoma pallidum antibody screening.

This study showed that the β-thalassemia trait is the most common hemoglobinopathy, with an overall prevalence rate of 2.59%. Our results are lower than the previously reported from the north and northeast of Iraq, where the majority of the population is Kurdish, with a carrier rate ranging from 3.7% to 4.14% [4,8,18]. It is also lower than reports from central and southern Iraq, where the majority of the population is Arab, with a reported carrier rate of 4.4% to 4.6% [4,19,20]. The reported carrier rate of β-thalassemia trait varies in the surrounding countries. For example, Turkey reported a frequency of 2% [21], while Iran reported a range of 4%-10% in various regions [22]. Saudi Arabia reported a frequency of 3.4% [23], while in Jordan, it was 3.3% [24]. Syria reported an overall prevalence of 7% [25].

Sickle cell trait was detected in 0.4% of the participants in the current study and found to be slightly higher than the previously reported rate of 0.27% from northern Iraq [18]. However, it is significantly lower than reports from the central and extreme south of Iraq, with a figure of 1.2%-6.5% [8,19]. Also, it is lower than the reported rate of most surrounding countries where a figure of 4.9% was reported in Turkey [21]. Saudi Arabia reported an average of 5.7% [23], and Iran reported an overall frequency of 1.5% [22]. Interestingly, no cases of HbC, HbD, or hereditary persistence of fetal hemoglobin (HPFH) were reported from the current study, which differs from some previous reports of Iraq [18,19].

Other than β-thalassemia minor and sickle cell trait, other hemoglobinopathies like δβ-thalassemia trait were not commonly detected in the current study with a reported figure of 0.076% of participants and found to be consistent with earliest studies in Iraq [8,18,19].

This study suggests the diagnosis of α-thalassemia is around 0.30%, which is likely underestimated in our region and across Iraq. The fact that single-gene deletion of the α-globin may present with normal or near-normal hematological and clinical features makes detection difficult without molecular studies. This may contribute to it is underestimation in the current study. While the majority of two and three-gene deletions are present with varied clinical and hematological data, ranging from asymptomatic with borderline low red cell indices to mild-moderate hypochromic anemia, and most of them need just observation, folic acid supplementation, and sometimes blood transfusion during stress events, like pregnancy or infection. However, α-thalassemia can often be confused with iron deficiency anemia, β-thalassemia trait, and δβ-thalassemia trait, necessitating an accurate interpretation of the red cell parameters, iron studies, HPLC, and family and genetic studies for diagnosis and confirmation.

Iron deficiency anemia was detected in 5.12% of cases, making it the most common cause of hypochromic microcytic anemia in this study. The majority of affected individuals were females. Although this rate is lower than previously reported by the WHO, it remains significant [26,27]. Since the majority of the studied population is in the young and middle-aged group, IDA imposes a considerable burden on the families and healthcare system, particularly during marriage and future pregnancy. IDA has a significant impact on future pregnancy outcomes, highlighting the importance of early detection and management [28].

Since there are no other screening programs for detecting hemoglobinopathy in this region, the mandatory premarital screening program for hemoglobinopathies plays a crucial role in detecting hemoglobinopathies among newly married couples. It effectively detects couples at risk of major hemoglobinopathies, aligning with the program's primary objective. However, the fact of high consanguineous rates (28.8% in this study), and the short time frame before marriage appointment (66.58% of couples arranged their marriage in less than two weeks, while 29.30% of the couples arranged their marriage within four weeks) make genetic counseling and separation of at-risk couples challenging and socially unacceptable. This justifies the need for population-based studies, particularly targeting families with high consanguineous rates, those with a genetic history of hemoglobinopathy, or those with a history of blood transfusion, to identify carriers before engagement and marriage.

Finally, the possibility of both silent β-thalassemia and a false-negative sickling test due to technical errors should always be taken into consideration. Strict observations of the test results, together with mandatory HPLC analysis for couples at risk or in question, especially among consanguineous marriages or those with a family history of hemoglobinopathies are strongly recommended.

This study has several limitations. The study primarily relies on hematological parameters and HPLC for diagnosis. More advanced molecular techniques, such as genetic sequencing, could provide a definitive diagnosis and detect silent or uncommon mutations. Genetic study of suspected α-thalassemia cases should be performed for accurate diagnosis and subclassifications according to the number of alpha gene deletions.

## Conclusions

The incidence of hemoglobinopathy is statistically low in this study but remains a significant health problem. Given the significantly high consanguinity marriage and the limited time until the wedding, population-based screening and screening among families with high consanguinity rates or those with a history of hemoglobinopathies are crucial.

Intensified premarital screening and counseling programs and careful interpretation of investigations with an urgent need to raise public awareness about the impact of consanguinity marriages through mass media campaigns and education programs can further decrease the incidence rate and mitigate their effects on public health.
